# Clinical experiences and accuracy of stereoelectroencephalography using the robotic arm Cirq

**DOI:** 10.1007/s00701-025-06541-4

**Published:** 2025-04-29

**Authors:** Kohei Kanaya, Asuka Nakamura, Daishiro Abe, Yutaro Sato, Mana Wakabayashi, Tomoya Shigehara, Daichi Watanabe, Yuki Yoshizawa, Tetsuhiro Fukuyama, Tetsuyoshi Horiuchi

**Affiliations:** 1 Department of Neurosurgery, Shinshu University School of Medicine, 3-1-1 Asahi, Matsumoto, 390-8621 Nagano Japan; 2 Epilepsy Center, Shinshu University Hospital, Matsumoto, Nagano Japan; 3 Department of Pediatrics, Shinshu University School of Medicine, Matsumoto, Nagano Japan; 4 Department of Clinical Engineer, Shinshu University Hospital, Matsumoto, Nagano Japan

**Keywords:** Stereoelectroencephalography, SEEG, Robot, Navigation, Cirq

## Abstract

**Background:**

Robot-assisted stereoelectroencephalography (SEEG) has become increasingly popular worldwide. Robotic arm Cirq (BrainLab, Munich, Germany) is an optional instrument for SEEG. This study aimed to evaluate the accuracy of electrode implantation using Cirq.

**Methods:**

Data were retrospectively collected from 10 consecutive SEEG cases from July 2022 to August 2024 at our institute. Two cases of simultaneous SEEG and grid implantation via craniotomy were excluded. Eight SEEG cases (37 depth electrodes) were included in this study. We evaluated the accuracy of the electrode placement. The distances between the planned and actual site of entry and the target were measured in the anterior-posterior (Xe, Xt) and cranial-caudal (Ye, Yt) directions. The distance between the planned and the actual target site was measured at the surface depth (Zt). The two-dimensional differences of the entry (De_2_) and target (Dt_2_) and the three-dimensional differences, including the depth parameter of the target (Dt), were measured. The two-dimensional and three-dimensional Euclidean distances (ED_2_, ED) were also calculated.

**Results:**

The differences between the planned entry and the actual entry in Xe and Ye were 2.5 ± 1.6 mm and -0.6 ± 1.8 mm, respectively. De_2_ was 3.2 ± 1.4 mm. The differences between the planned target and the actual target in Xt, Yt, and Zt were 2.1 ± 1.5 mm, 0.5 ± 1.5 mm, and 1.4 ± 2.9 mm, respectively. Dt_2_ and Dt were 2.7 ± 1.4 mm and 4.1 ± 1.7 mm, respectively. ED_2_ and ED were 1.8 ± 1.1 mm and 3.4 ± 1.8 mm, respectively.

**Conclusions:**

We reported our initial experience with a high accuracy and features of the Cirq robotic arm for SEEG procedures using the standard surface matching method.

## Introduction

Talairach and Bancaud developed stereoelectroencephalography (SEEG) using a stereotactic frame in France in the 1950s [2]. SEEG with depth electrodes has become common worldwide, particularly in European countries and the United States [1].

Recently, a navigation-guided robotic-arm system was developed with the aid of recent improvements in navigation systems. Robot-assisted SEEG has the advantage of reducing surgical time compared to SEEG using a stereotactic frame, and has an equivalent complication rate and accuracy [5]. There were several reports about the accuracy using several types of navigation-guided robotic arm systems such as the Stealth Autoguide (Medtronic, USA), ROSA (Zimmer Biomet, USA), Neuromate (Renishaw, UK) [3, 5, 7].

Recent reports showed the efficacy and safety of Cirq for cranial biopsies and SEEG [9, 10]. Truckenmueller et al. reported the high accuracy of the Cirq with automated registration, integrating robotic cone-beam CT [9]. The evaluation of the accuracy using newly navigation-guided robotic arm systems is important, however, there were few reports about the accuracy and the features of the robotic arm Cirq. Furthermore, there was no report about the accuracy of SEEG using Cirq with standard surface matching method. This study aimed to present our clinical experience of the accuracy and features using the robotic arm Cirq for SEEG with standard surface matching method.

## Material and methods

### Patients

The data were retrospectively collected from 10 consecutive SEEG cases from July 2022 to August 2024. Two cases of simultaneous depth electrode implantation using Cirq and grid implantation via craniotomy were excluded. Eight SEEG cases (37 depth and 370 contact electrodes) were included in this study. Two SEEG cases (16 depth electrodes and 158 contact electrodes) were bilateral depth electrode implantation.

### Preoperative planning and implantation of SEEG

**Fig. 1:**
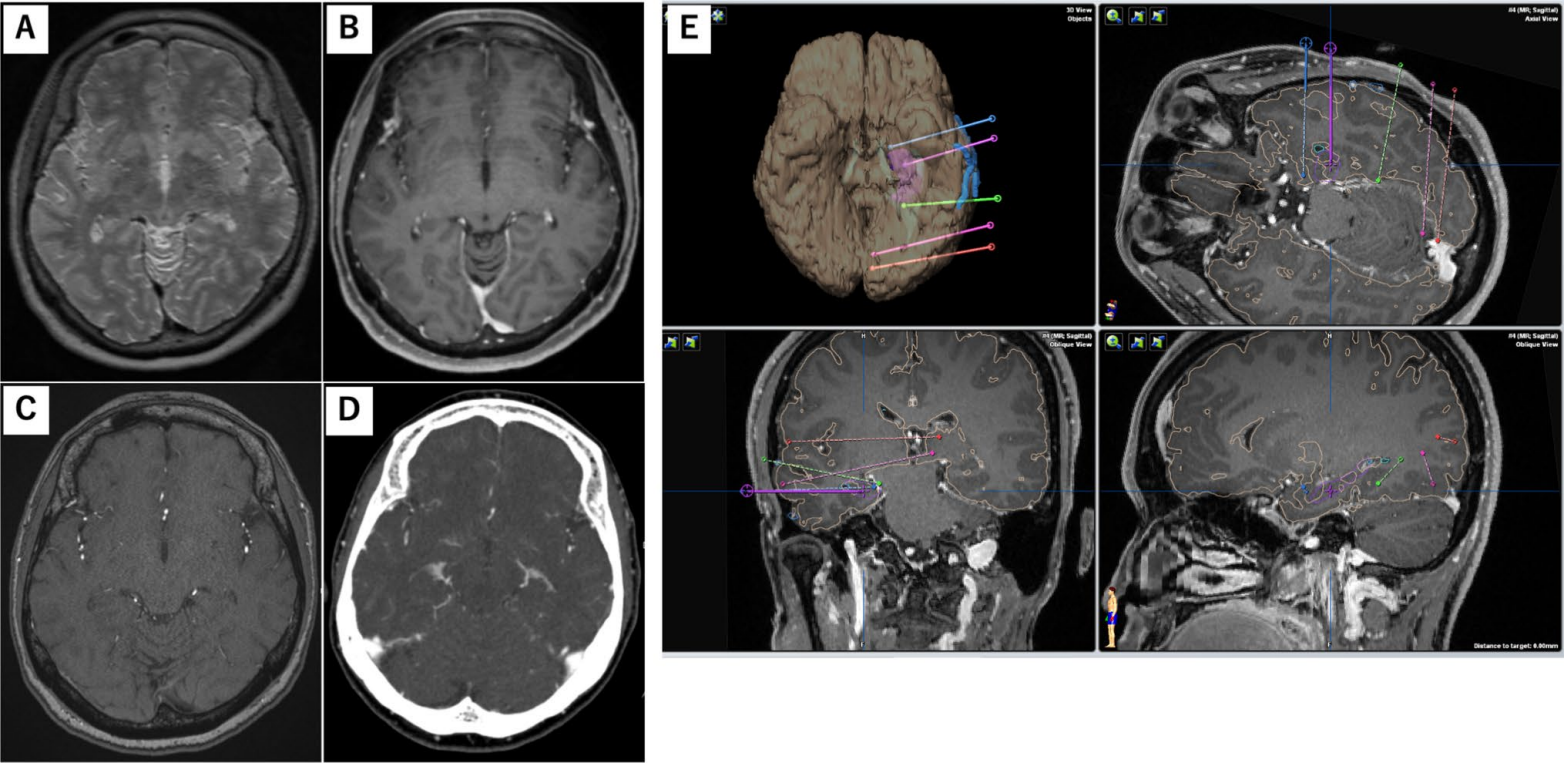
Preoperative planning of SEEG with T2 thin slice (**A**), enhanced T1 imaging (**B**), time of flight (**C**), and enhanced CT (**D**). SEEG planning and 3D reconstruction using iPlan (**E**)

Preoperatively, SEEG planning was performed using iPlan (BrainLab, Germany) based on thin images of T2, contrast-enhanced T1, time of flight MRA, and contrast-enhanced CT scans in venous phase (Fig. 1A-D). Contrast-enhanced CT was performed using a 256-detector row CT scanner. The scan parameters were as follows: tube voltage, 120 kV; tube current, 500 mA; matrix size, 512 × 512; field of view, 220–240 mm; and slice thickness, 0.625 mm. The venous phase was obtained 30–40 s after intravenous administration of iopamidol (370 mg/mL, 100 mL) at 3–4 mL/s. The trajectory was planned to be more than 5 mm from the vessels and sulcus. The angle between the trajectory and skull was greater than 60°, and the distance between the electrodes was greater than 10 mm as much as possible. The trajectories of all the electrodes were planned using iPlan (Fig. 1E).

**Fig. 2:**
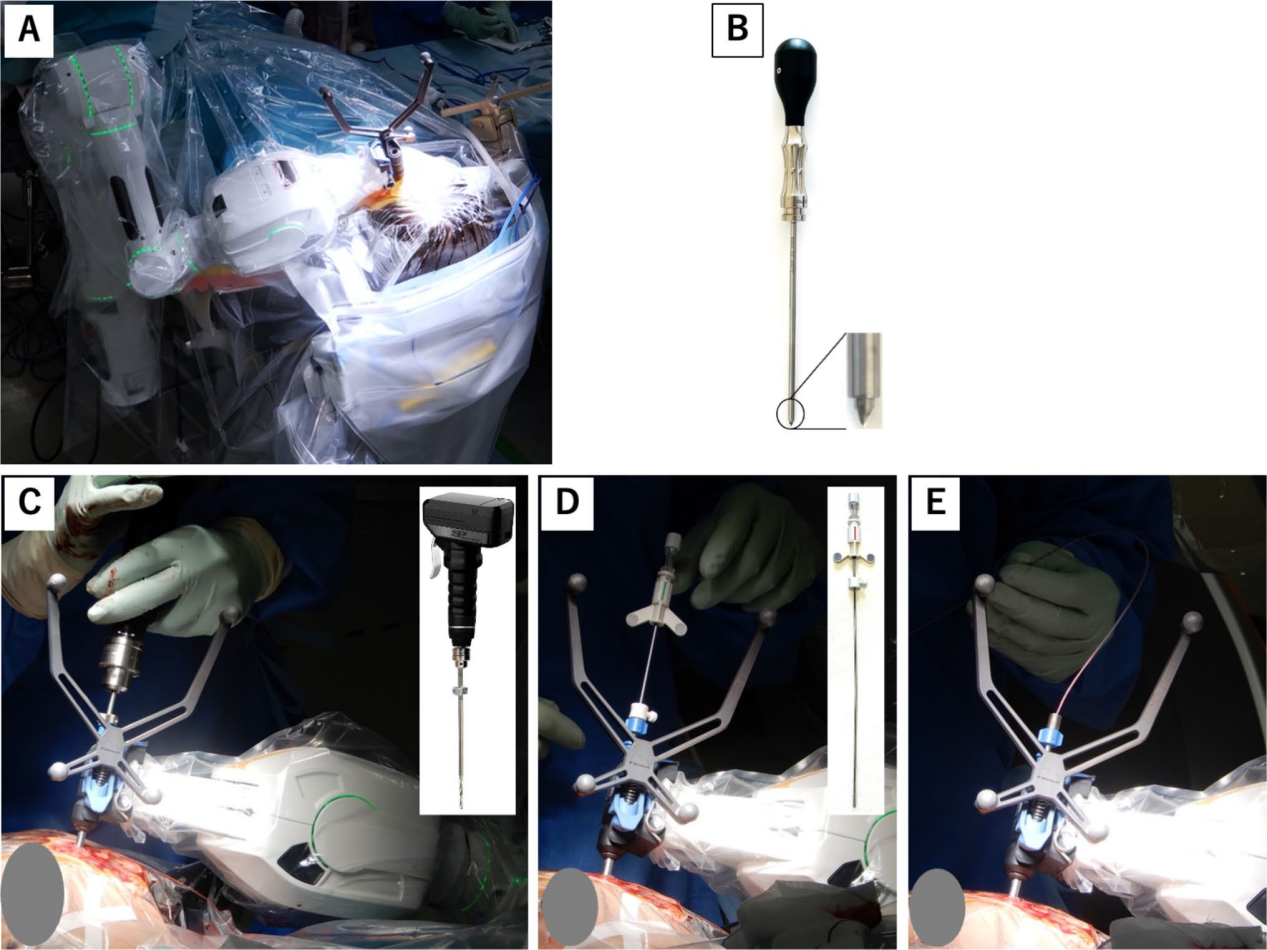
Robotic arm Cirq with bed rail (**A**). Indentation to the skull made using bone pick (**B**). Drilling of the skull using SP motor drill (**C**). Insertion of the biopsy needle into the target (**D**). Placement of the depth electrodes (**E**)

The robotic arm, Cirq, was set on the operative side rail (Fig. 2A). Under general anesthesia, the patient’s head was fixed using a radiolucent head frame. Registration of the navigation system using surface matching method (BrainLab, Germany) based on CT scan was performed and surgery was initiated. An approximately 2 cm skin incision was made, and the guide tube with Cirq was fixed to the skull. Before making a hole, a bone depression was made using a bone pick (S&Brain, Japan) to prevent the twist drill from slipping (Fig. 2B). A 2.4 mm skull hole was made using an SP motor drill (S&Brain, Japan) (Fig. 2C). The bone anchor in the guide tube was fixed on the skull, and then a 1.8 mm thick biopsy needle (Brainlab, Germany) was inserted to the target under the guidance of the navigation system to create the trajectory (Fig. 2D). Subsequently, a 1.5 mm thick depth electrode (Unique Medical, Japan) was implanted along the same trajectory. (Fig. 2E). The placement of the depth electrode was confirmed to match the same trajectory of the biopsy needle insertion, using X-ray imaging with the ARTIS Pheno (Siemens, Germany), which is a multi-axis X-ray fluoroscopy system. The electrodes were fixed to the scalp using sutures because the use of anchor bolts is not permitted in the Japanese healthcare system.

### Implanted electrodes evaluations

**Fig. 3:**
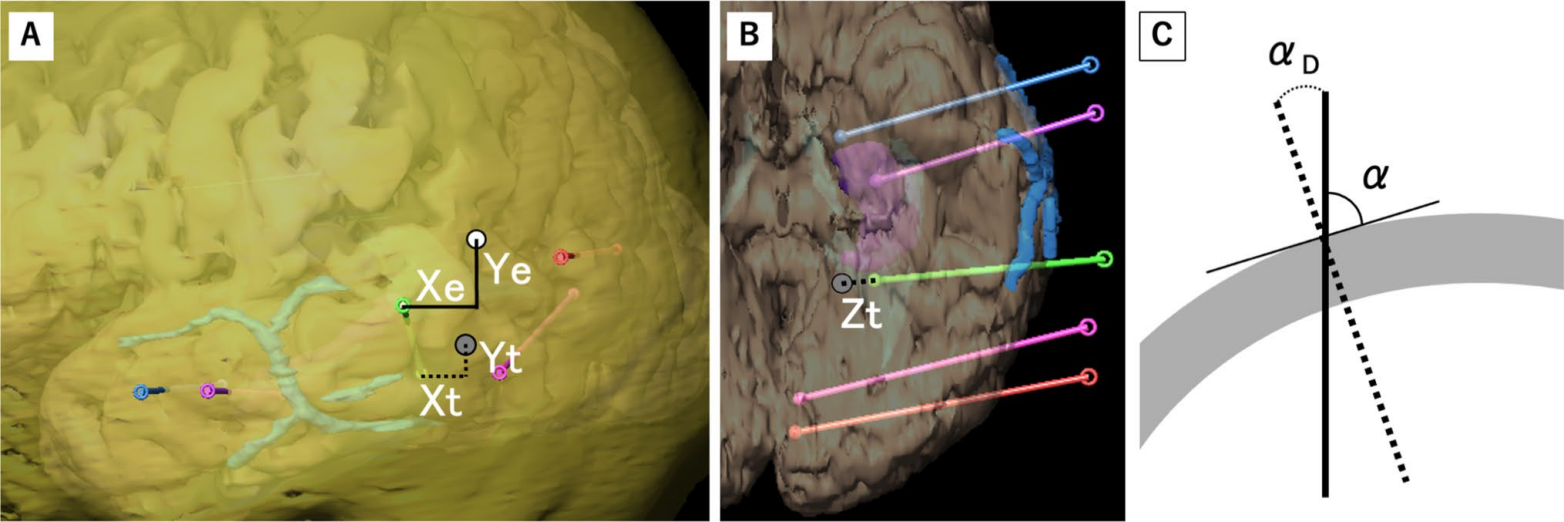
Analysis method of the differences between the planned and actual SEEG. The anterior-posterior differences at entry and target point showing Xe and Xt, and the cranial-caudal differences at entry and target point showing Ye and Yt (**A**). Differences of surface-depth at the target point showing Zt (**B**). The actual entry point (white circle) and actual target point (gray circle). Angle between the planned trajectory and skull (α). The differences between the planned and actual angle (α_D_). The solid line shows the planned trajectory. The dotted line shows the actual trajectory (**C**)

We evaluated the accuracy of electrode placement using postoperative CT scans compared to preoperative planning using iPlan (BrainLab, Germany). The distances between the planned and actual sites of entry and the target were measured in the anterior-posterior (Xe, Xt) and cranial-caudal (Ye, Yt) directions (Fig. 3A). The distance between the planned site and the actual target site of the implanted electrode was measured at the surface depth (Zt) (Fig. 3B). The difference of the entry point at the surface depth (Ze) was defined as zero. Posterior, cranial, and depth sides compared to the planned sites were defined as positive.

The two-dimensional differences of the entry (De_2_) and target (Dt_2_) were calculated using the following formulas: De_2_ = $\sqrt{{{\varvec{X}}{\varvec{e}}}^{2}+{{\varvec{Y}}{\varvec{e}}}^{2}}$ and Dt_2_ = $\sqrt{{{\varvec{X}}{\varvec{t}}}^{2}+{{\varvec{Y}}{\varvec{t}}}^{2}}$.

Three-dimensional differences of the target (Dt) were calculated using the following formula: Dt = $\sqrt{{{\varvec{X}}{\varvec{t}}}^{2}+{{\varvec{Y}}{\varvec{t}}}^{2}+{{\varvec{Z}}{\varvec{t}}}^{2}}$.

The two-dimensional Euclidean distance was calculated using the following formula: ED_2_ = $\sqrt{{({\varvec{X}}{\varvec{e}}-{\varvec{X}}{\varvec{t}})}^{2}+{({\varvec{Y}}{\varvec{e}}-{\varvec{Y}}{\varvec{t}})}^{2}}$.

Three-dimensional Euclidean distance was calculated using the following formula: ED = $\sqrt{{({\varvec{X}}{\varvec{e}}-{\varvec{X}}{\varvec{t}})}^{2}+{({\varvec{Y}}{\varvec{e}}-{\varvec{Y}}{\varvec{t}})}^{2}+{({\varvec{Z}}{\varvec{e}}-{\varvec{Z}}{\varvec{t}})}^{2}}$.

The angle between the planned trajectory and the skull was evaluated (α). The difference between the planned and actual angles was also measured (α_D_) (Fig. 3C).

We evaluated the following clinical characteristics of all patients: age, sex, etiology, side, target, Xe, Ye, De_2_, Xt, Yt, Zt, Dt_2_, Dt, ED_2,_ ED, α, and α_D._ Data are shown as mean ± standard deviation. Pearson correlation coefficients (r) and Bland-Altman analysis between Xe and Xt, Ye and Yt, and De_2_ and Dt_2_ were statistically analyzed. Bland-Altman analysis was shown as mean difference (95% lower and upper limits of agreement). Surgical time per electrode was also shown.

## Results

### Patients and electrodes

Characteristics of the patients and electrodes are shown in Table 1. A total of thirty-seven depth electrodes were used. No surgical complications were observed.

**Table 1:** Characteristics of the patients and electrodes

N.	Age, Sex	Etiology	Side	Target	Entry (mm)			Target (mm)							Angle (degree)	
					Xe	Ye	De2	Xt	Yt	Zt	Dt2	Dt	ED2	ED	α	α_D_
1	31, F	focal cortical dysplasia	L	amygdala	3.8	− 2.3	4.4	2.5	0	1.4	2.5	2.9	2.6	3.0	75.3	0.0
				hippocampus	2.3	− 2.7	3.5	0.3	2.7	6.1	2.7	6.7	5.8	8.4	87.4	1.8
				parahippcampal gyrus	3.4	− 3.1	4.6	3.4	− 1.3	1.6	3.6	4.0	1.8	2.4	83.8	5.0
3	30, F	hippocampal sclerosis	L	amygdala	2.4	0	2.4	2.4	0.6	3.8	2.5	4.5	0.6	3.8	86.1	4.4
		occipital lobe epilepsy		hippocampus	3.8	0.5	3.8	2.7	1.4	3.5	3.0	4.6	1.4	3.8	89.6	0.9
				parahippcampal gyrus	4.1	− 1.4	4.3	2.7	− 1	0.5	2.9	2.9	1.5	1.5	71.6	0.0
				Lingal gyrus	3.9	− 0.2	3.9	4.3	0	2.1	4.3	4.8	0.4	2.1	52.3	0.7
				Cuneus	3.2	− 1.2	3.4	1.7	− 0.3	7.4	1.7	7.6	1.7	7.6	54.4	0.0
4	58, F	hippocampal sclerosis	L	amygdala	0.6	− 0.4	0.7	− 0.3	0.6	− 0.3	0.7	0.7	1.3	1.4	84.4	6.4
				hippocampus	0.2	− 1.4	1.4	− 0.3	0.1	1.8	0.3	1.8	1.6	2.4	85.9	0.0
				parahippcampal gyrus	0.8	− 0.9	1.2	0.8	0.4	4.5	0.9	4.6	1.3	4.7	72.7	7.6
5	30, M	focal cortical dysplasia	R	amygdala	3.8	2	4.3	0.7	0	1.3	0.7	1.5	3.7	3.9	87.8	3.6
				hippocampus	3.8	− 2.2	4.4	2.9	− 1.9	0.4	3.5	3.5	0.9	1.0	84.0	5.2
				parahippcampal gyrus	2.5	− 2.5	3.5	0.6	0	2.2	0.6	2.3	3.1	3.8	77.3	11.2
6	75, F	hippocampal sclerosis	L	amygdala	0	0.3	0.3	− 0.7	1.8	3.9	1.9	4.4	1.7	4.2	73.9	0.1
				hippocampus	0.1	0.5	0.5	− 0.7	1	3.6	1.2	3.8	0.9	3.7	74.1	5.6
				parahippcampal gyrus	0.4	2.1	2.1	0.8	2	1.4	2.2	2.6	0.4	1.5	87.2	6.8
8	54, M	bil. hippocampal sclerosis	R	amygdala	2.2	− 3.1	3.8	2.5	0	0	2.5	2.5	3.1	3.1	73.2	5.0
				hippocampus	3	− 2	3.6	2.9	− 0.3	2.8	2.9	4.0	1.7	3.3	84.6	2.2
				parahippcampal gyrus	2.6	− 3.3	4.2	3.9	0	0	3.9	3.9	3.5	3.5	87.7	0.9
				posterior temporal	4.5	− 4	6.0	4.4	− 1.9	0	4.8	4.8	2.1	2.1	70.6	0.3
			L	amygdala	3.2	1.4	3.5	3.9	3	− 2.1	4.9	5.3	1.7	2.7	77.0	0.9
				hippocampus	4.4	2.1	4.9	4.1	3.6	2.9	5.5	6.2	1.5	3.3	90.0	2.1
				parahippcampal gyrus	4.2	1.4	4.4	4.4	3.9	1.2	5.9	6.0	2.5	2.8	88.9	9.7
9	20, M	postencephalitis epilepsy	R	amygdala	3.5	0.8	3.6	2.4	0.8	0.7	2.5	2.6	1.1	1.3	83.9	3.2
		bil. hippocampal sclerosis		hippocampus	4.2	0.6	4.2	3.1	0	− 3.6	3.1	4.8	1.3	3.8	86.6	2.1
				parahippcampal gyrus	3.8	1.1	4.0	4.2	0.9	− 2.1	4.3	4.8	0.4	2.1	73.3	3.7
				posterior temporal 1	3.1	2.7	4.1	2.7	2.6	1.3	3.7	4.0	0.4	1.4	68.5	11.8
				posterior temporal 2	3.5	2.2	4.1	1.3	2.7	0.8	3.0	3.1	2.3	2.4	81.5	8.1
				parietal	4.9	1.3	5.1	1.8	1.2	4.7	2.2	5.2	3.1	5.6	86.0	11.6
			L	amygdala	0	− 2.7	2.7	1.4	− 1.7	− 0.5	2.2	2.3	1.7	1.8	85.5	3.2
				hippocampus	0	− 1.3	1.3	2	− 0.9	− 7.6	2.2	7.9	2.0	7.9	85.0	0.6
				parahippcampal gyrus	0.3	− 2.5	2.5	3	− 0.8	− 4	3.1	5.1	3.2	5.1	81.3	10.7
10	51, M	mutliple hemangiomas	L	amygdala	0.4	− 2.7	2.7	0	− 1	0.9	1.0	1.3	1.7	2.0	79.0	1.0
				hippocampus	1.5	− 1.4	2.1	0.4	− 1.2	4	1.3	4.2	1.1	4.2	86.2	2.0
				parahippcampal gyrus	1.2	− 1.2	1.7	1.7	0.5	5.9	1.8	6.2	1.8	6.2	71.5	0.6
				orbital gyrus	1.2	0	1.2	2.1	− 0.5	3	2.2	3.7	1.0	3.2	77.0	0.0
				Agerage	2.5	− 0.6	3.2	2.1	0.5	1.4	2.7	4.1	1.8	3.4	79.6	3.8
				SD	1.6	1.8	1.4	1.5	1.5	2.9	1.4	1.7	1.1	1.8	8.8	3.7

Differences among individual electrodes were evaluated

*F *female, *M *male, *L *left, *R *right, *SD *standard deviation

The differences between the planned entry and the actual entry in Xe and Ye were 2.5 ± 1.6 mm and − 0.6 ± 1.8 mm, respectively. De_2_ was 3.2 ± 1.4 mm.

The differences between the planned target and the actual target in Xt, Yt, and Zt were 2.1 ± 1.5 mm, 0.5 ± 1.5 mm, and 1.4 ± 2.9 mm, respectively. Dt_2_ and Dt were 2.7 ± 1.4 mm and 4.1 ± 1.7 mm, respectively. ED_2_ and ED were 1.8 ± 1.1 mm and 3.4 ± 1.8 mm, respectively.

The angle between the planned trajectory and skull (α) was 79.6 ± 8.8°. The difference in the angles between the planned trajectory and the actual trajectory (α_D_) was 3.8 ± 3.7°.

The Pearson correlation coefficients (r) between Xe and Xt, Ye and Yt, and De_2_ and Dt_2_ were 0.629 (*p* <.001), 0.739 (*p* <.001), and 0.619 (*p* <.001), respectively. Bland-Altman analysis showed that Xe-Xt was 0.400 (− 2.067- 2.867), Ye-Yt was − 1.095 (− 3.687- 1.498), and De_2_-Dt_2_ was 0.546 (− 1.831- 2.923).

The average surgical time per electrode was 30.9 minute (range: 25.8–46.3 minute).

### Representative case of bilateral SEEG using robotic arm Cirq

**Fig. 4:**
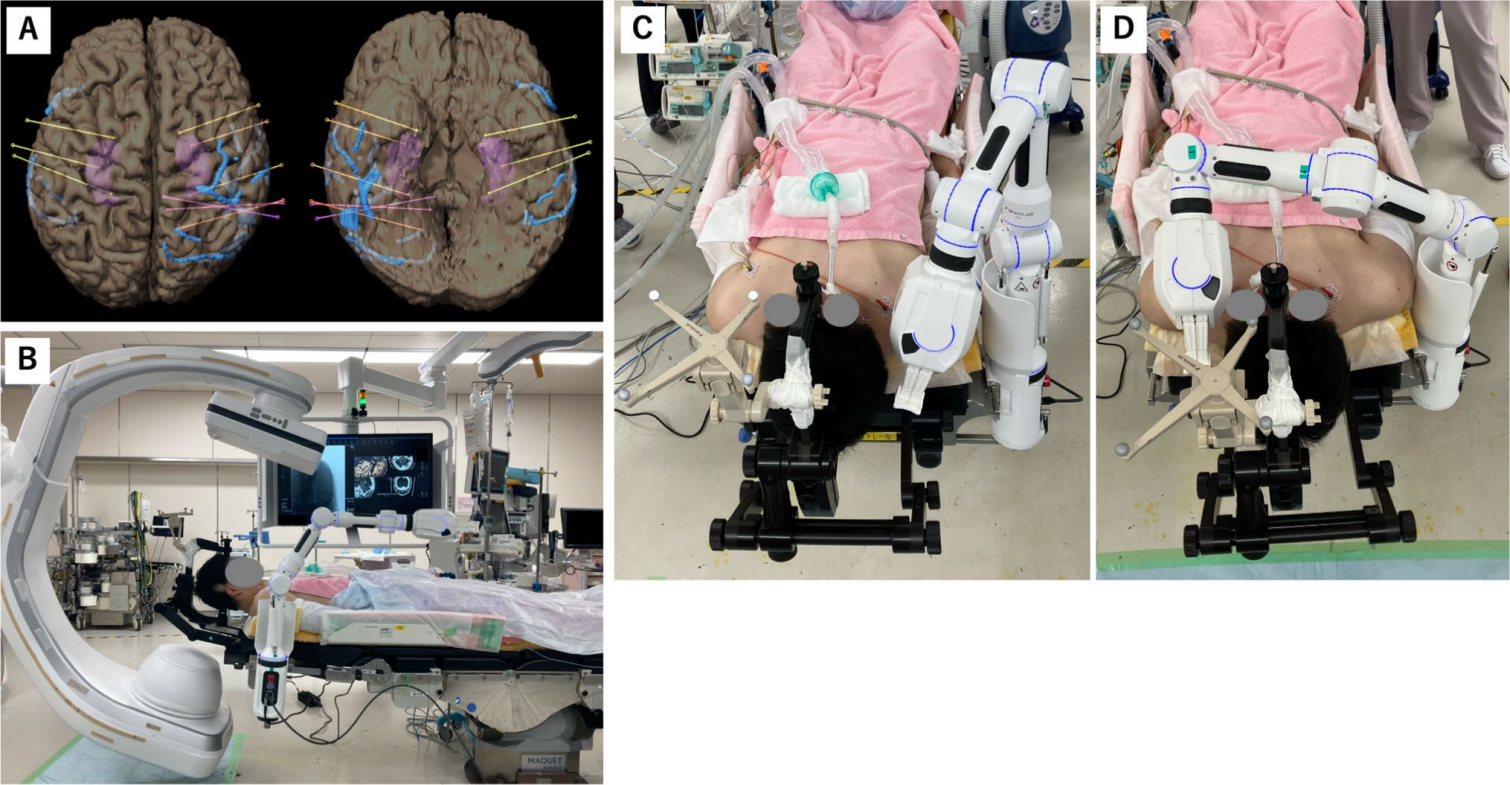
Representative case of bilateral SEEG and SEEG planning using iPlan (**A**). Operative view with robotic arm Cirq and ARTIS Pheno (**C**). Robotic arm Cirq fixed with bed rail at right side reaching right (**C**) and left (**D**) hemispheres

A 20-year-old man with a history of autoimmune encephalitis at age 12 presented with intractable post-encephalitic epilepsy, refractory to four antiseizure medications. He experienced two distinct types of habitual seizures: one characterized by visual blurring in the left visual field, progressing to a tonic-clonic seizure with left-sided predominance, and another beginning as a focal impaired awareness seizure that evolved into a tonic-clonic seizure with right-sided predominance. Ictal video-electroencephalography suggested epileptogenic foci in the right posterior temporal and parietal lobes, as well as in the left temporal lobe. To localize the epileptogenic zones, bilateral SEEG was planned, with six depth electrodes placed in the right posterior temporal and parietal regions and three in the left temporal lobe (Fig. 4A). His head was fixed in a neutral position using Doro and a skull cramp holder. The robotic arm Cirq was fixed with a bed rail, and ARTIS Pheno was prepared (Fig. 4B). Cirq could reach the bilateral hemispheres; therefore, bilateral SEEG could be performed without head or Cirq re-fixation. (Fig. 4C, D). Depth electrodes were implanted on both sides as usual.

## Discussion

This study demonstrates the accuracy and the safety of SEEG using the robotic arm Cirq with standard surface matching method. Our data showed that the differences in the entry point were positively related to those in the target point. Therefore, it was important to make an accurate entry as planned, especially the registration of the navigation system, which may be most important for the accuracy about the entry and the target point.

The accuracy of the navigation system is influenced by both the imaging modality and the registration method employed. Spyrantis et al reported that CT-frame referencing and CT-laser-based referencing offer superior accuracy compared to 3.0-T MRI-laser-based referencing [8]. Accordingly, at our institution, navigation system registration is performed using CT imaging. While we currently utilize the standard surface registration method, greater accuracy may be achieved through automatic intraoperative registration using the ARTIS Pheno system, as described by Truckenmueller et al. [9]. Similarly, Grote et al. demonstrated the high accuracy and effectiveness of automatic intraoperative CT-based registration for the implantation of SEEG depth electrodes [6]. These findings suggest that automatic intraoperative registration may offer improved precision over conventional surface registration techniques.

The difference in the depth parameter can relate to the fixation method of the depth electrodes. The electrodes were fixed with nylon sutures on the scalp at our institute because anchor bolts are not permitted in the Japanese healthcare system. Our data show that the difference between Dt_2_ and Dt, which is a difference in the depth parameter, was 1.4 mm on average. The use of an anchor bolt provides a more accurate SEEG of the depth parameter.

Cardinale et al. reported similar accuracy using a robot-assisted device (NeuroMate, Reinshaw, United Kingdom) compared with that of the Talairach frame-based method. The median target error was 1.77 mm (interquartile range, 1.25–2.51; range, 0.08–15.40 mm) using a robot-assisted device in 81 SEEG procedures with 1050 electrodes [3]. Gonzalez-Martinez et al. reported a similar accuracy (median target error, 1.7 mm; interquartile range, 1.2–2.3 mm) with their 101 robot-assisted (ROSA, Medtech, France), frameless SEEG procedures [5]. Kojima et al. reported that the mean entry error and the mean target error using Stealth Autoguide (Medtronic, Minneapolis, MN, USA) were 1.99 ± 0.90 mm and 3.59 ± 2.22 mm in two SEEG procedures with 17 electrodes [7]. Our data showed that the accuracy of SEEG using Cirq was similar to that of other devices, although data with a large number of SEEG may be better than our data [3, 5, 7]. Our data in this study were a small number and initial experience, as Kojima et al. reported [7]; however, the accuracy will improve as the number of cases increases and surgical skill improves. Furthermore, the use of an anchor bolt can improve the accuracy of SEEG. Truckenmueller et al. reported the mean entry error and the mean target error were 1.4 ± 1.2 mm and 2.6 ± 1.6 mm using Cirq with automatic intraoperative registration of the navigation system in five SEEG procedures [9]. Automatic intraoperative registration can improve the accuracy of SEEG rather than the standard surface matching method.

The main reason for the differences in navigation-guided SEEG is misalignment of the registration of the navigation system. Other factors may be a slip of the drill, peeling of the dura until dural penetration, fixation of the electrodes, postoperative swelling of the scalp and muscle in suture fixation, and brain shift due to CSF leakage during surgery. The steep angle to the skull can be related to the slipping of the drill and the difference in direction. Therefore, we used a bone pick to depress the skull and prevent slipping of the drill tip when drilling. The drill tip can be steadied to the skull after depressing the skull using a bone pick.

SEEG has become increasingly popular worldwide. Robot-assisted SEEG offers the advantage of reduced surgical time compared to SEEG performed with a stereotactic frame [5]. In our experience, the surgical time per electrode was comparable to that reported by Kojima [7]. This duration may further decrease with ongoing technical advancements.

Navigation-guided robotic arm systems such as the Stealth Autoguide (Medtronic, USA), ROSA (Zimmer Biomet, USA), Neuromate (Renishaw, UK), and Cirq (BrainLab, Germany) are commonly used for SEEG. The Stealth Autoguide was used for fixation with the head frame. The ROSA and Neuromate are floor-based robotic arms. The robotic arm Cirq is used not only for neurosurgical but also orthopedic surgeries, especially in spinal surgeries [4], because the robotic arm Cirq is fixed on the operative bed and has seven degrees of freedom and a wide range. It is easy to handle and reach the head for SEEG, even bilaterally. In bilateral SEEG procedures, avoiding the need for surgical repreparation including head refixation and re-registration of the navigation system can save approximately 30 minutes to an hour.

This study had several limitations. First, the study had a small sample size from a single institute. Second, several devices are unavailable in Japan, such as anchor bolts and thin electrodes. Despite these limitations, this study highlighted the accuracy and features of the robotic arm Cirq for SEEG. Furthermore, the differences in our data will improve as the number of SEEG cases increases and technical improvements are made. To the best of our knowledge, there have been no clinical studies on the robotic arm Cirq for SEEG using surface registration method, and our preliminary data may be helpful for the usage of the robotic arm Cirq.

## Conclusion

We report our initial experiences with the accuracy, safety, and features of the robotic arm Cirq for SEEG. Accurate registration of the navigation system is most important for a precise SEEG. Further improvements are expected in the registration method, skull drilling, and electrode fixation for more accurate SEEG.

## Data Availability

No datasets were generated or analysed during the current study.

## References

[1] Abou-Al-Shaar H, Brock AA, Kundu B, Englot DJ, Rolston JD (2018) Increased nationwide use of stereoencephalography for intracranial epilepsy electroencephalography recordings. J Clin Neurosci 53:132–134. 10.1016/j.jocn.2018.04.064 29724650 10.1016/j.jocn.2018.04.064 PMC6188665

[2] Bancaud J, Angelergues R, Bernouilli C et al (1970) Functional stereotaxic exploration (SEEG) of epilepsy. Electroencephalogr Clin Neurophysiol 28(1):85–86 4188481

[3] Cardinale F, Cossu M, Castana L et al (2013) Stereoelectroencephalography: surgical methodology, safety, and stereotactic application accuracy in 500 procedures. Neurosurgery 72(3):353–66. 10.1227/NEU.0b013e31827d1161 23168681 10.1227/NEU.0b013e31827d1161

[4] Chesney K, Triano M, Dowlati E, Zhang I, Felbaum DR, Aulisi EF (2022) Cirq robotic arm-assisted transpedicular instrumentation with intraoperative navigation: technical note and case series with 714 thoracolumbar screws. J Robot Surg 16(4):893–898. 10.1007/s11701-021-01313-5 34606045 10.1007/s11701-021-01313-5

[5] González-Martínez J, Bulacio J, Thompson S et al (2016) Technique, results, and complications related to robot-assisted stereoelectro-encephalography. Neurosurgery 78(2):169–180. 10.1227/NEU.0000000000001034 26418870 10.1227/NEU.0000000000001034

[6] Grote A, Gjorgjevski M, Carl B et al (2025) Frameless stereotaxy in stereoelectroencephalography using intraoperative computed tomography. Brain Sci 15(2):184. 10.3390/brainsci15020184 40002517 10.3390/brainsci15020184 PMC11853342

[7] Kojima Y, Uda T, Kawashima T et al (2022) Primary experiences with robot-assisted navigation-based frameless stereo-electroencephalography: higher accuracy than neuronavigation-guided manual adjustment. Neurol Med Chir (Tokyo) 62(8):361–368. 10.2176/jns-nmc.2022-0010 35613881 10.2176/jns-nmc.2022-0010 PMC9464478

[8] Spyrantis A, Cattani A, Woebbecke T et al (2019) Electrode placement accuracy in robot-assisted epilepsy surgery: a comparison of different referencing techniques including frame-based CT versus facial laser scan based on CT or MRI. Epilepsy Behav 91:38–47. 10.1016/j.yebeh.2018.11.002 30497893 10.1016/j.yebeh.2018.11.002

[9] Truckenmueller P, Früh A, Kissner JF et al (2024) Integration of a lightweight and table-mounted robotic alignment tool with automated patient-to-image registration using robotic cone-beam CT for intracranial biopsies and stereotactic electroencephalography. Neurosurg Focus 57(6):E2. 10.3171/2024.9.FOCUS24525 39616638 10.3171/2024.9.FOCUS24525

[10] van Baarsen KM, Woodley DEA, Slot KM, Woerdeman PA, Han KS, Hoving EW (2024) Robotic alignment system Cirq (Brainlab) for navigated brain tumor biopsies in children. Childs Nerv Syst 40(1):99–108. 10.1007/s00381-023-06060-6 37436473 10.1007/s00381-023-06060-6

